# The Sex Determination Gene Shows No Founder Effect in the Giant Honey Bee, *Apis dorsata*

**DOI:** 10.1371/journal.pone.0034436

**Published:** 2012-04-12

**Authors:** Zhi Yong Liu, Zi Long Wang, Wei Yu Yan, Xiao Bo Wu, Zhi Jiang Zeng, Zachary Y. Huang

**Affiliations:** 1 Honeybee Research Institute, Jiangxi Agricultural University, Nanchang, China; 2 Experimental Animal Center, Institute of Occupational Disease Prevention, Nanchang, China; 3 Department of Entomology, Michigan State University, East Lansing, Michigan, United States of America; 4 Ecology, Evolutionary Biology and Behavior Program, Michigan State University, East Lansing, Michigan, United States of America; Ghent University, Belgium

## Abstract

**Background:**

All honey bee species (*Apis spp*) share the same sex determination mechanism using the complementary sex determination (*csd*) gene. Only individuals heterogeneous at the *csd* allele develop into females, and the homozygous develop into diploid males, which do not survive. The honeybees are therefore under selection pressure to generate new *csd* alleles. Previous studies have shown that the *csd* gene is under balancing selection. We hypothesize that due to the long separation from the mainland of Hainan Island, China, that the giant honey bees (*Apis dorsata*) should show a founder effect for the *csd* gene, with many different alleles clustered together, and these would be absent on the mainland.

**Methodology/Principal Findings:**

We sampled *A. dorsata* workers from both Hainan and Guangxi Provinces and then cloned and sequenced region 3 of the *csd* gene and constructed phylogenetic trees. We failed to find any clustering of the *csd* alleles according to their geographical origin, i.e. the Hainan and Guangxi samples did not form separate clades. Further analysis by including previously published *csd* sequences also failed to show any clade-forming in both the Philippines and Malaysia.

**Conclusions/Significance:**

Results from this study and those from previous studies did not support the expectations of a founder effect. We conclude that because of the extremely high mating frequency of *A. dorsata* queens, a founder effect does not apply in this species.

## Introduction

In honey bees (genus *Apis*), as in other hymenopteran insects, sex is controlled by a mechanism named complementary sex determination (*csd*) [1], [2]. Diploid heterozygotes become females, while haploids develop into males. When the two copies of *csd* alleles are homozygous, the individual develops into a diploid male, which is eaten by workers [3]. Therefore, honey bees are under great selective pressure to become heterozygous. This complementary sex determination theory remained a hypothesis until recently. Beye et al. [4] cloned the *csd* gene by positional cloning and RNA interference. They found that this gene maintains many highly variable alleles in a group and no transcription difference exist between the sexes. *Csd* encodes an arginine serine-rich (SR) type protein, which contains an RS domain in the middle and a proline-rich region at its C terminus, between these two domains is a hypervariable region that differs highly among alleles and has variable numbers of asparagines/tyrosine repeats. The honey bee *csd* is homologous to *Drosophila tra*, which is involved in *Drosophila* sex determination.

**Figure 1:**
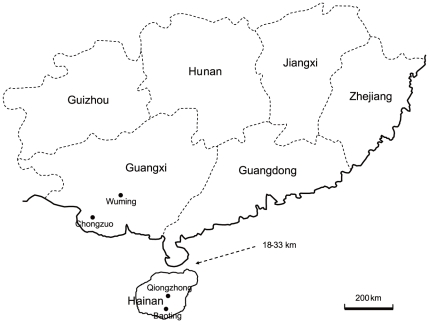
A map showing the strait between Hainan Island and Guangxi Province and the geographical distribution of the collected samples in this study.

Previous studies have shown that *csd* is evolving under balancing selection, and several parts of the coding region are possible targets of selection [5]–[9]. Moreover, the polymorphic level is approximately seven times higher in *csd* than in the neutral regions [8]. Although a previous study found no “bottleneck” effect in the *csd* gene of *A. dorsata* and *A. mellifera*, but suggestive of such an effect in *A. cerana* [9], the Hainan Island in China provides a more specific opportunity to test whether the *csd* gene has a founder effect. Hainan Island in China was thought to have been separated from the mainland about 20 million years ago [10]. The width of the Qiongzhou Strait separating Hainan Island from the mainland is about 18–33 km (Fig. 1). The maximum distance of *Apis mellifera* queen mating fight is about 15 km [11], but most of the queens mated at a distance of 7.5 km or less.

It was suggested that *Apis dorsata* drones have “significantly shorter drone flight distance” compared to *A. mellifera* [12]. Regardless, the 18 km distance of the narrowest distance in the straight should be far enough to prevent any gene exchange between the *A. dorsata* populations in the island and the mainland. It is believed that the *csd* gene is under balancing selection, that is, it is under positive selection to create more alleles to avoid becoming homozygous, but at the same time alleles are lost due to sampling effect, creating a balancing force. If the *A. dorsata* population on Hainan Island has been separated from these on the mainland for more than 20 million years, we expect that due to the founder effect [13], the island should have many different alleles of *csd* compared to the mainland due to the initial sampling and then the expansion of the initial population. In addition, the island population should be under stronger selection force due to a smaller initial subset of the mainland *csd* alleles. This can be manifested by showing a higher nucleotide diversity value, due to either a higher level of polymorphism or a higher mutation rate. In this study we tested whether island *A. dorsata* populations experience the founder effect and stronger positive selection by comparing *csd* genes in workers sampled on the island and those on the mainland.

## Methods

### Samples collection

*A. dorsata* samples were collected from two provinces of China, Hainan Province, and Guangxi Province. The Hainan and Guangxi provinces are geographically separated by the Qiongzhou Strait. In each province, three *A. dorsata* colonies were collected, with 30 adult workers from each colony. In Hainan, two colonies were sampled from Qiongzhong County and one colony from Baoting County; and in Guangxi, two colonies were sampled from Chongzuo County and one from Wuming County. The *A. dorsata* in China are feral and usually nest on tall trees, adult workers were coll**e**cted from colonies nested in trees using a net after climbing up the tree. The samples were first collected into 95% ethanol and then stored at −70°C until use.

### DNA extraction

Total genomic DNA was extracted from the head and thorax of each sampled bee according to the protocol of the Animal Genomic DNA Extraction Kit (BEST ALL-HEAL LLC, NY, USA)

### PCR and sequencing

The primers used for amplifying region 3 of *A. dorsata csd* gene in this study were designed by consulting those reported by [8] with some modifications. The primers are 5′ AATTGGATTTATTAATATAATTTATTATTCAGG 3′ (forward) and 5′ ATYTCATTATTCAATATGTTNGCATCA 3′ (reverse). High Fidelity LA Tag DNA polymerase (BEST ALL-HEAL LLC, NY, USA) was used for all PCR reactions. PCR conditions were denaturation at 94°C for 3 min, followed by 30 cycles of 94°C for 30 s, annealing at 54°C for 30 s and extension at 72°C for 2 min, with a final extension at 72°C for 7 min. PCR products were purified by using DNA GEL EXTRACTION kits (BEST ALL-HEAL LLC, NY, USA) and cloned into pEASY-T3 vectors (Transgene, Beijing, China). To obtain as many *csd* alleles as possible, the genomic region 3 of *csd* gene was cloned from each sample, and 1–3 clones of each cloned fragment were subjected to double-strand sequencing. Single-sequencing reads were assembled by using Seqman program in DNAstar software [14].

### Definition of csd alleles

To avoid the effect of neutral mutation on our results, we adopted the method used by Hasselmann et al. [9], we classified those sequence variants that form a bush-like structure in the tree as neutral variants of a single allele. In each neutral cluster, only one sequence was chosen as an allele for further analysis, while the remaining sequences of the same cluster were excluded. We identified these clusters by π estimates and Z test on the whole genome sequences as well as the coding sequences of these alleles. Similar to Hasselmann and Beye [9], we found that using this method, sequences classified as neutral variants had the same repeat structure in the hyhpervariable region, while those identified as alleles were unique in that region.

### Sequence analysis

Sequences of *A. dorsata csd* genomic region 3 (N = 42 sequences) published by other research groups were downloaded from Genbank under accession numbers DQ325038 - DQ325060 and EU100917- EU100935, repetitive sequences were removed and the remaining sequences (N = 31) were used for phylogenetic analysis together with our sequences. Exons, introns and coding regions of these sequences were determined by consulting those sequences of *csd* genomic region 3 reported by [8]. Nucleotide sequence alignments were performed with Clustal X version 1.8 [15], the alignment results were adjusted manually for obvious alignment errors.

DAMBE 4.1.19 [16] was used to identify haplotypes. Phylogenetic trees were constructed using MEGA version 4.0 program [17]. The minimal evolution (ME) method and Kimura's 2-parameter distances were adopted to obtain an unrooted tree with 2,000 bootstrap replications. Nucleotide diversity (π) and gene flow value (Nm) were calculated by using DNAsp5.0 program [18]. Two tailed Z test was used to detect significant differences between π values of Guangxi and Hainan populations. Pair-wise Fst distances were computed using ARLEQUIN 3.1 software [19]. Jost D test was performed at the website based program SMOGD with 200 bootstrap replicates [20]. Median joining (MJ) network was drawn using the program Network 4.5 [21].

## Results

### *csd* variation in Guangxi and Hainan populations

For the *csd* gene region 3, we obtained a total of 63 and 55 different sequence variants from Guangxi and Hainan populations, respectively. These 63 and 55 sequence variants were respectively aligned and genealogy trees were constructed. After removing neutral variants, 17 different alleles from Guangxi and 18 different alleles from Hainan province were chosen for further analysis. Of them, 5 alleles were shared by both Guangxi and Hainan populations, resulting in a total of 30 haplotypes. To verify that our sequences were indeed *csd* gene, we compared our sequences with those *Apis dorsata csd* allele sequences reported by Hasselmann *et al.* [9] through amino acid sequence alignment (Figure S1). The alignment results showed that our sequences were highly similar to those *csd* alleles reported by Hasselmann et al. [9]. For example, each of our alleles contained a hypervariable region between the arginine/serine-rich (RS) domain and the proline rich domain, just as reported in [9]. The hypervariable region was rich in asparagine (N) and tyrosine (Y) that form (N)_1–4_Y repeats and (KHYN)_1–4_ repeats in each allele.

The nucleotide diversity values (π) of *csd* in Guangxi and Hainan populations were 0.06123±0.00576 and 0.05271±0.00631, respectively, without a significant difference between the two (two tailed Z test, Z = 0.997, P>0.05, Table 1). Moreover, the nucleotide diversity values of coding region, exon region and intron region of *csd* alleles also showed no significant differences between these two locations (Table 1).

**Table 1:** Nucleotide diversity (π) (mean ± SD) of *csd* alleles in Hainan and Guangxi populations.

populations	Genome	Coding region	Exons	Introns
GX	6.123±0.576	5.777±0.560	6.921±0.642	4.435±0.599
HN	5.271±0.631	5.278±0.702	5.510±0.633	5.417±1.246

### Genetic differentiation between Guangxi and Hainan populations

The population pairwise Fst genetic distance between Guangxi and Hainan populations was 0.00236. The gene flow value (Nm) between these two populations was 10.71. The harmonic mean of D_est_ value between them was −0.03.

### Phylogeny of *Apis dorsata csd* alleles

**Figure 2:**
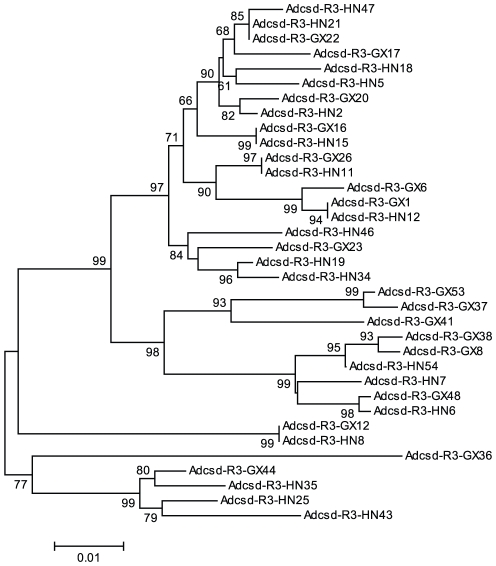
The gene genealogy of *csd* alleles in region 3 from Guangxi and Hainan *A. dorsata* populations. The minimum evolution method and Kimura's two parameter distances were used to construct the tree. Bootstrap percentages are shown on internal branches. The scale bar represents the number of nucleotide changes per site. Geographical origin is indicated by letters: HN, Hainan; GX, Guangxi.

**Figure 3:**
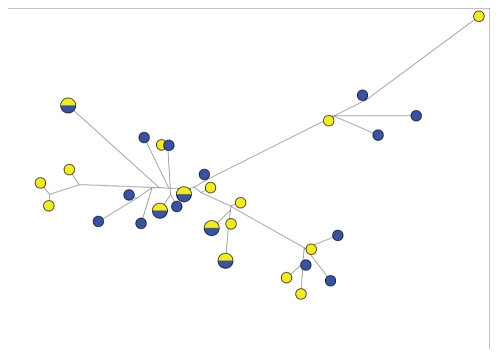
The median joining network of 30 haplotypes in Guangxi and Hainan populations. Circles denote haplotypes unique to Guangxi (yellow), or unique to Hainan (blue), or in both Guangxi and Hainan (half yellow and half blue). The sizes of circles are proportional to haplotype frequencies.

**Figure 4:**
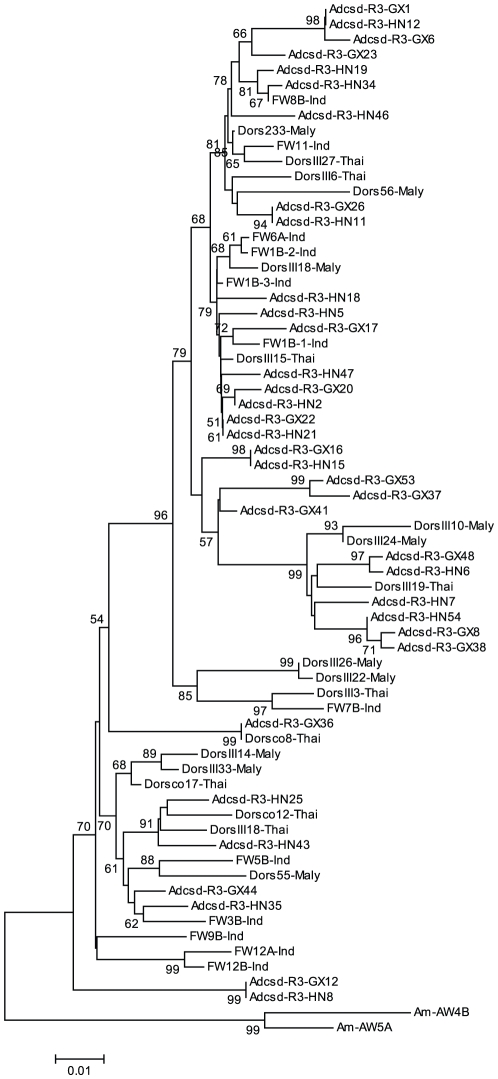
The gene genealogy of region 3 of the *A. dorsata csd* alleles from this study and those from Cho et al. 2006 [**8**] and Hasselmann et al. 2008 [**9**]. The coding sequences of all the alleles were used. The minimum evolution method and Kimura's two parameter distances were used to construct the tree. Bootstrap percentages are shown on internal branches. The scale bar represents the number of nucleotide changes per site. Amcsd-AW4B and Amcsd-AW5A, both from the Western honey bee *Apis mellifera*, were used as outgroup sequences.

When all the alleles form Guangxi and Hainan populations were used to construct a phylogenic tree by using the ME method, *csd* alleles did not form two branches reflecting the Guangxi and Hainan samples. Rather, the two groups were well mixed among each other (Fig. 2). A median joining network was constructed based on the 30 haplotypes to investigate the possible relationships among these haplotypes (Fig. 3). The network suggests that the haplotypes from the same population varied greatly, and haplotypes from different geographical populations were intermixed. We then constructed another tree using ME method, using the coding region of all the available *csd* data for *A. dorsata*, including these obtained in our current study, and that from [8] and [9] (Fig. 4). Again, it showed very little or no clustering according to geographical origins of each *csd* allele.

## Discussion

Contrary to our original hypothesis, we did not find an expansion of *csd* alleles in *Apis dorsata* at the Hainan province, which would manifest as a different clade in a phylogenetic tree (Fig. 2). Furthermore, after we combined sequence data from our study and previous studies [8], [9], we failed to find any clustering of *A. dorsata csd* alleles from other islands such as the Philippines and Malaysia. The separation times of these two regions from the mainland were believed to be 23 million and 40 million years, respectively [22]. Our results are consistent with a previous study by Hasselmann et al. [9] showing that the *csd* genes of *A. mellifera*, and *A. dorsata* not showing a bottleneck effect. Further, their *csd* geneaology tree (Fig. 2) showed a similar intermix of *csd* alleles originating from Malaysia and Thailand.

The Fst distance between the Hainan and Guangxi *A. dorsata* populations was 0.236%, that is, 99.764% of genetic variation was within populations and 0.236% was among populations. In general, Fst<5% indicates a low level of genetic differentiation [23]. There was also a high gene flow value (Nm = 10.71) between these two populations, suggesting frequent exchange of genetic material between these two populations in history. All these results demonstrated that the genetic differentiation between these two populations was very low.

The lack of geographical clustering of *A. dorsata* alleles and the small genetic difference between the Hainan and Guangxi populations can be explained by two possible hypotheses. One is that our original assumption of the separation time between Hainan and the mainland was not accurate. Indeed, the separation time of Hainan from the mainland remains a mystery. While [10] cited a separation time of 20 million years, others claim the separation was not stable and the straight was dried and refilled many times since then [24]. The most recent separation was believed only to be 15,000 years ago. If the second estimate is correct, then we do not expect any *csd* changes in such a short time frame.

The alternative hypothesis is that even if the separation time is indeed 20 million years, one still would not expect a founder effect in the *csd* gene due to the particularity of honey bee biology. This is due to the highly polyandrous nature of honey bees, especially for *A. dorsata*. Previous reports for the queen mating frequency is 20–26 times for *A. dorsata* [25], [26], but a more recent study reported that the maximum number of subfamilies found per colony is 102 and the effective mating frequency is 88.5 [27]. The total number of *csd* alleles world wide is probably well under 100, and in a particular region is more likely to be about 20–30 ([5]–[7], this study). Therefore, even if only one colony of *A. dorsata* was “sampled” to be on the Hainan island during the separation (which is a highly unlikely low number), there would still be enough *csd* alleles preserved in the island to prevent the occurrence of any founder effect. This is confirmed by results of analysis after including the *csd* data from other island like countries, such as the Philippines and Malaysia (Fig. 4).

Our current study therefore suggests that due to the highly polyandrous nature of honey bees, the founder effect does not apply to the *csd* gene in *Apis dorsata*, perhaps also to any other gene, to most *Apis* species. Consistent with this idea, a previous study [28] on Africanized bees in Costa Rica also showed that “that Africanized honey bee populations are very representative of the genetic variation of African bees from the Transvaal region of South Africa.” In contrast, in social insects that have no multiple matings, such as the fire ant *Solenopsis invicta*, a founder effect was found in their sex determination system [29].

## Supporting Information

Figure S1 **Amino acid sequence alignment of region 3 of the** ***Apis dorsata csd*** **alleles from this research (those started with Adcsd) and those reported by Hasselmann** ***et al.*** **2008** [**9**] **(those started with Dors).** A total of 54 predicted amino acid sequences were used. Alignment was done by Clustal X version 1.8 software. The alignment suggests that our sequences from DNA are truly *csd* alleles because they are structurally highly similar to those of [9] which were from cDNA. Both our sequences and those from Hasselmann et al. [9] contain the arginine/serine-rich (RS) domain, the hypervariable region, and the proline rich domain. Furthermore, number 53 (this study) and number 54 [9] are identical in all amino acid sequences. Others, such as 29, 30, 31 only show small differences (2 in RS domain, 1 in proline rich domain), other than the differences in the hypervariable region.
(EPS)
